# Plaque Reduction Efficacy of an Advanced Electric Toothbrush Compared with a Manual Toothbrush Among 6‐ to 10‐Year‐Old Children: Results From a 4‐Week Randomised Controlled Trial

**DOI:** 10.1111/ipd.70086

**Published:** 2026-04-28

**Authors:** Avi Zini, Lital Zecharyahu, Shirya Teitelbaum, Renzo Alberto Ccahuana‐Vasquez, Hans Timm, Julie Grender, Esti Davidovich

**Affiliations:** ^1^ Faculty of Dental Medicine Hebrew University & Hadassah Jerusalem Israel; ^2^ Procter & Gamble Service GmbH Kronberg Germany; ^3^ The Procter & Gamble Company Mason Ohio USA

**Keywords:** dental plaque, electric toothbrush, oral hygiene, oscillating‐rotating, paediatrics, randomised controlled trial

## Abstract

**Background:**

An advanced oscillating‐rotating (OR) electric toothbrush was developed to help improve plaque control for paediatric patients.

**Aim:**

To assess plaque reduction efficacy of an advanced OR electric toothbrush compared with a manual toothbrush among children.

**Design:**

This was a 4‐week randomised, controlled, two‐treatment, parallel‐group, examiner‐blind clinical study. Sixty children, 6–10 years of age, were randomly assigned to a test group: 1) Advanced OR electric toothbrush (Oral‐B iO2 with Gentle Care brush head) or a control group: 2) Manual toothbrush (Paro Junior Soft). Both groups brushed twice daily with a fluoridated dentifrice. Plaque was assessed at baseline and 4 weeks using the Turesky modification of the Quigley‐Hein Plaque Index. Data were analysed with ANCOVA.

**Results:**

Sixty participants were enrolled (30/group); mean (SD) age 7.7 (±1.33) years. Fifty‐eight children completed the trial. At week 4, the test group had 51% greater whole mouth plaque reduction compared with the control group (0.67 versus 0.44; p = 0.003). The test group also had statistically significantly greater plaque reductions (*p* ≤ 0.021) than the control group in lingual, buccal, approximal, and molar subregions, ranging from 42%–64% greater.

**Conclusion:**

The advanced OR electric toothbrush produced significantly greater plaque reductions compared with a manual toothbrush among a paediatric population.

## Why this paper is important to paediatric dentists

- Poor oral health leading to childhood caries is a problem worldwide and has been found to be a predictor of caries and periodontal disease in adults.
- Developing effective oral hygiene habits, with an emphasis on toothbrushing, early in life has been shown to reduce the risk of these diseases in adulthood.
- Key to good oral hygiene is mechanical plaque control via effective toothbrushing, and results of this 4‐week study among children 6–10 years old provide evidence that an advanced OR electric toothbrush reduced plaque more effectively than a manual toothbrush and could assist with the goal of achieving better oral health among children.

## 1 Introduction

The poor oral health of children worldwide has been well documented in the literature [1, 2]. Findings from a recent systematic review and meta‐analysis comprised of 164 articles, conducted by Kazeminia et al. [1], reported the prevalence of dental caries globally to be 46.2% for the primary dentition and 53.8% for the permanent dentition. With a combined sample size of over 1.5 million children, these results provide significant evidence for the global need to elevate the oral health status of children and are consistent with World Health Organization (WHO) data [1, 2]. Furthermore, while the introduction of fluoride in various sources (e.g., water, salt, milk, oral hygiene products) has reduced caries levels, access is not universal and more than 500 million children worldwide still have cavities; it is the most common non‐communicable disease globally [1, 2].

Fortunately, effective oral hygiene strategies can reduce plaque levels and help prevent caries. The use of a toothbrush is the most important tool for daily oral hygiene and plaque control. However, good oral hygiene habits must include not only the recommended frequency (2×/day) and duration of toothbrushing (2 min per brushing) [3] but also the thoroughness of brushing, given that 95% of deciduous caries are in the molar region [4]. Research shows the importance of establishing these good self‐care habits early in life, as children with poor plaque control have been reported to lose four times more teeth by their early 30s due to cavities than children categorised as having better plaque control [5].

Typically, the majority of children use a manual toothbrush [6]. However, recent studies have shown that use of an electric toothbrush significantly improves children's oral health [7, 8, 9, 10, 11, 12]. In two separate systematic reviews and meta‐analyses of randomised and controlled paediatric clinical trials, electric toothbrushes were shown to be more effective than manual toothbrushes for plaque removal in children [10, 11]. Although there has not been a systematic review comparing different electric toothbrush technologies for plaque removal among children, there is abundant clinical evidence for oscillating‐rotating (OR) electric technology [7, 8, 9, 10, 11]. In a separate cross‐sectional evaluation of dental records for 1000 children, those using an OR electric toothbrush showed 6 times greater odds of less plaque and 1.4 times greater odds of being caries‐free compared with children using a manual toothbrush [12]. These findings indicate that changing from a manual to an electric toothbrush, particularly an oscillating‐rotating model, is an opportunity to improve plaque control for children, thereby promoting better oral health.

Recently, an advanced OR electric toothbrush was introduced—Oral‐B iO Kids—developed with guidance from paediatric dental professionals, for children 6 years of age and older. The marketed toothbrush has a simple design and compliance‐enhancing features (e.g., characters, 2‐min music timer) to appeal to children. The aim of this investigation was to evaluate the plaque reduction benefits of the toothbrush model (without the compliance‐enhancing features of the music timer or colourful handle featuring a character that could potentially impact results) compared with a manual toothbrush among children. This is the first clinical evaluation reported for this electric toothbrush among a paediatric population. A manual toothbrush was chosen as the comparator since it is commonly used by children.

## 2 Materials and Methods

### 2.1 Ethics Approval

Before study initiation, the Investigator obtained institutional review board (IRB) ethics approval from Hadassah Medical Center IRB committee (Jerusalem, 91120, Israel; Ref: HMO 0401‐24). The trial was conducted in compliance with the protocol and in accordance with the ethical principles of Good Clinical Practice, according to the International Council for Harmonization Harmonized Tripartite Guideline and all applicable regulatory requirements.

### 2.2 Study Participants

Sixty (60) children were screened and enrolled in the study as all met the inclusion criteria of: being between the ages of 6 and 10 years; being in good general health; possessing a minimum of 16 natural teeth with facial and lingual scorable surfaces; and having evidence of dental afternoon plaque. Exclusion and continuance criteria are listed in Table 1.

**TABLE 1:** Participant exclusion and continuance criteria.

Exclusion criteria	Participants were excluded from study participation if they had: A disease or condition that might interfere with examination procedures or the ability of the participant to safely complete the study Urgent dental treatment needs Fixed orthodontic appliances Use of antibiotics within 2 weeks before study initiation A dental prophylaxis within 1 month before study initiation
Continuance Criteria	Participants could be excluded from the study or the analysis if they: A disease or condition that might interfere with examination procedures or the ability of the participant to safely complete the study Participated in any other oral care study Took any antibiotics or anti‐inflammatory medication during the trial Had a breach of study criteria or protocols for oral hygiene and pre‐visit food restrictions Did not have a parent or legal guardian present at all study visits

### 2.3 Study Design & Plan

This was a single‐centre, examiner‐blind, 4‐week, 2 treatment, parallel group, randomised study conducted from January 2025 to February 2025 at the the Department of Community Dentistry Faculty of Dental Medicine, Hadassah ‐ Hebrew University Medical Center in Jerusalem, Israel. At the Baseline visit, sixty children who met study entrance criteria, and a parent or guardian, signed the IRB consent form. Plaque measurements were taken by the same calibrated examiner at two time points: Baseline and Week 4 using the Turesky Modified Quigley‐Hein Plaque Index (TQHPI) [13, 14]. Any emergency treatment required during the study was to be referred to the local dental clinic for immediate treatment, and any study adverse events were documented.

Participants were asked to refrain from all oral hygiene procedures after their morning brushing (which was to be no later than 8 am) and to refrain from eating, chewing gum, or drinking for 3 h before the Baseline and Week 4 visits (small sips of water were allowed up to 45 min before the visit). Both visits were scheduled for approximately the same time in the afternoons.

#### 2.3.1 Visit 1 (Baseline)

Personal medical history and demographic information were collected and documented. Participants received an oral examination, and a red disclosing solution (Mira‐2‐Ton; Hager & Werken, Germany) was applied with cotton swabs on all teeth to stain for presence of plaque. The examiner then conducted the pre‐brushing plaque examination (TQHPI).

Participants meeting all inclusion criteria were enrolled and, along with their parents, proceeded to a protected area that ensured blinding of the examiner to the identity of the test products. Then participants were stratified by their baseline plaque scores (≤ 4.0 vs. > 4.0) and gender using a block size of 4. Within strata, participants were randomised to one of two treatment groups by site staff according to the encoded randomisation/balance and assignment program that the study sponsor created and provided to the site:

- Electric toothbrush group: Oral‐B iO2 OR electric toothbrush with Oral‐B iO Gentle Care brush head (OP030/OR017).
- Manual toothbrush group: Paro Junior Soft manual toothbrush.

Participants and their parents were given brushing instructions (both written and verbal) for their assigned toothbrush and marketed toothpaste (Colgate *Junior*, sodium monofluorophosphate, 1450 ppm F). Those in the electric toothbrush group were given manufacturer's instructions (twice daily brushing for two minutes per brushing). Those in the manual group were instructed to brush in their customary manner twice a day. Participants brushed their teeth with their assigned products under supervision by site staff unaided by a mirror.

Participants were scheduled for their 4‐week follow‐up visit, at approximately the same time of day as the Baseline visit. Parents were reminded that their children must follow the oral hygiene and eating/drinking restrictions before the visit.

Any adverse events (AEs) were recorded.

#### 2.3.2 Visit 2 (Week 4 Follow‐Up)

Participants returned to the site and brought their test products to return. Continuance criteria were assessed and recorded. Their teeth were disclosed and a TQHPI exam was conducted by the examiner. Any General Comments or AEs were recorded.

### 2.4 Turesky Modified Quigley‐Hein Plaque Index

The plaque deposits on the teeth were scored on six sites (distobuccal, midbuccal, mesiobuccal, distolingual, midlingual, and mesiolingual) of all deciduous and permanent teeth (excluding surfaces with restorations and malformations) according to the Quigley‐Hein Index as modified by Turesky et al. [13, 14]. Plaque scores range from 0 (no plaque) to 5 (plaque covering 2/3 or more of the side of the crown of the tooth). Whole mouth, buccal, lingual, approximal, and molar (defined as 1st and 2nd permanent molar) average plaque scores were calculated for each participant and tooth surfaces by totalling the scores and dividing by the number of gradable sites examined.

### 2.5 Statistical Analysis

Study size was determined to be a minimum of 28 participants per treatment group completing the study which should have provided at least 80% power to show a 0.356 significant treatment difference for whole mouth TQHPI 4‐week change from baseline with a standard deviation of 0.467, using two‐sided testing at a 5% significance level.

Summary statistics including means, standard deviations, frequencies, etc. of the demographic and baseline characteristics were calculated across all participants and for each treatment group.

Statistical analyses for plaque efficacy over the 4‐week product use were based on whole mouth average pre‐brushing TQHPI change from baseline scores (Baseline minus Week 4). Treatment differences for the 4‐week pre‐brushing plaque changes from baseline were assessed using an analysis of covariance (ANCOVA) with Baseline TQHPI as the covariate. The interaction between the covariate and treatment was also evaluated. Additionally, the 4‐week plaque changes were analysed for significance from zero for each treatment group using a paired difference *t*‐test.

Interproximal plaque scores, plaque scores on the lingual and buccal surfaces, and plaque scores on molars were also analysed separately within treatment and across treatments as specified above.

Four‐week whole mouth TQHPI change from baseline was the primary variable. All statistical tests were two‐sided and carried out at the 5% significance level.

### 2.6 Safety Evaluation

Safety was assessed by the absence of observed or self‐reported irreversible AEs.

## 3 Results

### 3.1 Participant Disposition and Demographics

**FIGURE 1:**
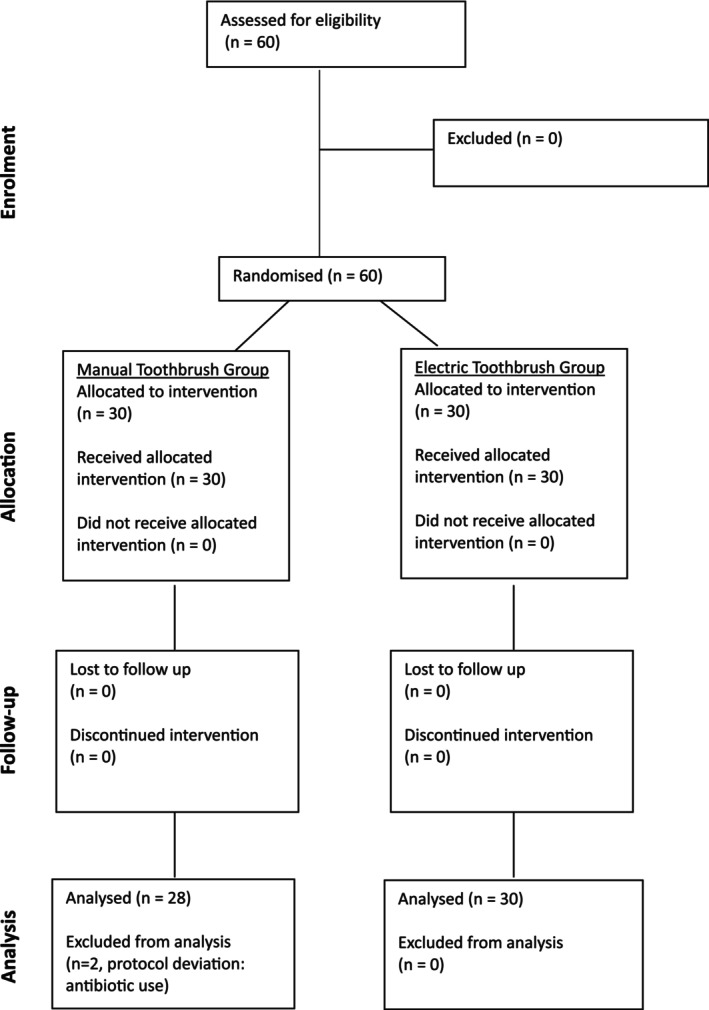
Flow diagram showing progression of participants in clinical trial.

Sixty participants were enrolled (30 per group). Data for two participants in the manual toothbrush group were excluded in the final analysis due to antibiotic use between the Baseline and Week 4 visit. See Figure 1. Treatment groups at baseline were balanced for demographic characteristics (*p* ≥ 0.848) and baseline (pre‐brushing) TQHPI scores (whole mouth, lingual, buccal, approximal, and molar region; all *p* ≥ 0.252). See Table 2. The population had a mean (SD) age of 7.7 (1.33) years, with a range of 6–10 years. 57% were female and 43% were male.

**TABLE 2:** Demographic summary and baseline plaque scores.

Characteristic	Manual toothbrush (*n* = 30)	Electric toothbrush (*n* = 30)	Overall (*n* = 60)	*p*‐value
Age (Years)				
Mean (SD)	7.7 (1.32)	7.7 (1.36)	7.7 (1.33)	0.848^†^
Min.—Max.	6–10	6–10	6–10	
Sex				
Female^‡^	17 (56.7%)	17 (56.7%)	34 (56.7%)	1.000^§^
Male^‡^	13 (43.3%)	13 (43.3%)	26 (43.3%)	
Race				
Black^‡^	0 (0.0%)	1 (3.3%)	1 (1.7%)	1.000^§^
Caucasian^‡^	30 (100.0%)	29 (96.7%)	59 (98.3%)	
TQHPI Score, mean (SD)				
Whole mouth	4.053 (0.2189)	4.026 (0.2070)	4.039 (0.2114)	0.622^†^
Lingual	3.768 (0.2840)	3.764 (0.2569)	3.766 (0.2679)	0.965^†^
Buccal	4.339 (0.2408)	4.287 (0.2128)	4.312 (0.2263)	0.384^†^
Approximal	4.089 (0.2168)	4.076 (0.1924)	4.082 (0.2028)	0.813^†^
Molar	3.825 (0.2357)	3.753 (0.2226)	3.787 (0.2296)	0.252^†^

^†^ Two‐sided ANOVA *p*‐value for the treatment comparison.

^‡^ The number (percent) of participants in each category.

^§^ Two‐sided Fisher's exact test *p*‐value for the treatment comparison.

### 3.2 Dental Plaque Evaluation (TQHPI)

Both toothbrushes delivered a statistically significant reduction from Baseline at Week 4 (*p* < 0.001) for whole mouth plaque and for all subregion plaque analyses (lingual, buccal, approximal, and molar). The whole mouth plaque reduction (percentage reduction vs. baseline) was 0.670 (16.6%) for the electric toothbrush, and 0.444 (11.0%) for the manual toothbrush. Subregion plaque reductions from Baseline at Week 4 ranged from 10.8% to 11.4% for the manual toothbrush and 15.6% to 17.8% for the electric toothbrush.

The electric toothbrush delivered a statistically significantly greater plaque reduction compared with the manual toothbrush for whole mouth plaque (0.670 vs. 0.444; 51.0%, *p* = 0.003) as well as plaque in lingual (0.670 vs. 0.408; 64.3%, *p* < 0.001), buccal (0.674 vs. 0.475; 41.8%, *p* = 0.021), approximal (0.673 vs. 0.442; 52.4%, *p* = 0.002), and molar (0.615 vs. 0.431; 42.8%, *p* = 0.011) subregions. See Table 3.

**TABLE 3:** Change from baseline efficacy results^†^ at 4 weeks: analysis of covariance summary^‡^.

Treatment	*N*	Overall Baseline Mean	Mean 4‐Week Score	Adjusted Mean (SE) Change from Baseline / % Change	2‐sided *p*‐value^‡^	Treatment Difference (SE)	Treatment Comparison 2‐sided *p*‐value 95% CI	% Treatment Difference Relative to Manual^§^
Whole Mouth								
Manual	28	4.04	3.60	0.444 (0.0518) 11.0%	< 0.001	−0.227 (0.0721)	0.003 (−0.371, −0.082)	
Electric	30	4.04	3.37	0.670 (0.0501) 16.6%	< 0.001			51.0%
Lingual								
Manual	28	3.77	3.36	0.408 (0.0526) 10.8%	< 0.001	−0.262 (0.0731)	< 0.001 (−0.409, −0.116)	
Electric	30	3.77	3.10	0.670 (0.0508) 17.8%	< 0.001			64.3%
Buccal								
Manual	28	4.31	3.84	0.475 (0.0602) 11.0%	< 0.001	−0.199 (0.0839)	0.021 (−0.367, −0.030)	
Electric	30	4.31	3.64	0.674 (0.0581) 15.6%	< 0.001			41.8%
Approximal								
Manual	28	4.08	3.64	0.442 (0.0510) 10.8%	< 0.001	−0.231 (0.0710)	0.002 (−0.374, −0.089)	
Electric	30	4.08	3.41	0.673 (0.0493) 16.5%	< 0.001			52.4%
Molar								
Manual	28	3.79	3.36	0.431 (0.0504) 11.4%	< 0.001	−0.184 (0.0698)	0.011 (−0.325, −0.044)	
Electric	30	3.79	3.18	0.615 (0.0477) 16.3%	< 0.001			42.8%

^†^ Change from Baseline = Baseline—Final.

^‡^ ANCOVA model included baseline and treatment effects. Equal treatment variances were modelled.

^§^ Percent treatment difference relative to manual = −100 * (Treatment Difference/Adjusted Mean of manual).

### 3.3 Safety Evaluation

There were no adverse events observed or reported in this study. Both treatments were well tolerated.

## 4 Discussion

This investigation is the first to evaluate the plaque reduction benefits of this OR electric toothbrush model compared with a manual toothbrush among children between 6 and 10 years of age. Both toothbrushes showed statistically significant plaque reductions over the 4‐week trial; however, the electric toothbrush provided a statistically significant 51% greater reduction in whole mouth plaque as well as greater reductions in all subregions, ranging from 42% to 64%.

These results are not surprising given that OR electric toothbrush technology has consistently demonstrated superior plaque removal and gingivitis efficacy, with a plethora of studies ranging over 2 decades, for both adults as well as children when compared with manual toothbrushes [7, 8, 9, 10, 11, 12, 15, 16, 17, 18]. The OR model tested in this trial has shown significantly greater plaque and gingivitis reductions compared with manual and sonic toothbrush control toothbrushes among adults in multiple randomised controlled trials [19, 20, 21]. This trial expanded upon those learnings to assess plaque effects among children. The model tested in this clinical trial was solid white; it did not have the aesthetic features of the marketed Kids' version (e.g., colourful handle, popular character, 2‐min music timer), which are intended to enhance brushing engagement and compliance. The effects of those features on compliance and plaque outcomes could be a topic for a future study.

The practical implications of these findings are important, as the trajectory of poor oral health in children could be significantly improved by use of a paediatric electric OR toothbrush with demonstrated plaque removal efficacy. An epidemiological study by Broadbent and colleagues [8] followed participants from 5 to 32 years of age and determined that individuals appear to follow a predictable pathway with oral health. A correlation was seen between a high plaque score at age 5 with a higher likelihood and severity of caries, more severe gingivitis, and increased tooth loss by age 32. While electric toothbrushes typically have a higher price point than manual toothbrushes, the data show that enhancing plaque control represents a valuable investment in the future oral health of patients who find them affordable. Moreover, electric toothbrush models for children are usually priced in the lower range of the overall electric toothbrush market to make them more affordable for more children to use.

With the knowledge that electric toothbrushes outperform manual toothbrushes among children [7, 8, 9, 10, 11, 12], and given that the majority of children still use a manual rather than an electric toothbrush [6], exploration of factors associated with this behaviour in addition to accessibility and affordability need further investigation. Unfortunately, there is a paucity of studies addressing this important issue. One qualitative study investigated the acceptability of an oral health behaviour change intervention that included evidence‐based resources (e.g., an OR electric toothbrush) and training to support oral health conversations between dental teams and parents [22]. Focus group findings indicated parents preferred the OR electric toothbrush for their children over the manual brush. It has been shown that habits such as toothbrushing are acquired through observational learning and modelling [23]. This suggests that perhaps children would be more likely to use an electric toothbrush if parents also purchased and used one.

A potential limitation of this trial is that it did not assess the effects of the OR brush on gingivitis, but research with advanced OR toothbrushes among adults consistently shows benefits for both plaque and gingivitis [16, 19, 20, 21], and gingivitis benefits have been demonstrated with a traditional OR model in a 4‐week paediatric trial [9]. Gingival health assessments could be included in future research, along with evaluations of the new OR toothbrush compared with other electric toothbrush models (e.g., traditional OR or sonic) for children. Key strengths of the clinical trial include use of a randomised controlled study design and a well‐established plaque index. Future qualitative studies on patient preferences and factors associated with behaviour change may help raise awareness of the positive benefits of early childhood interventions with electric toothbrushes on their overall oral health by creating life‐long habits.

In conclusion, an advanced OR electric toothbrush provided superior plaque reduction relative to a manual brush over a period of 4 weeks in children aged 6–10 years.

## Author Contributions

R.A.C.‐V., J.G., E.D., and A.Z. designed the study; R.A.C.‐V. served as a clinical scientist and medical monitor; H.T. served as a clinical trial manager; L.Z. served as the laboratory director and study coordinator; A.Z. served as a principal investigator; E.D. served as co‐investigator; S.T. served as an examiner; J.G. led the data analysis; R.A.C.‐V., J.G., A.Z., and E.D. interpreted the data; R.A.C.‐V. and J.G. participated in preparing the manuscript; A.Z., L.Z., S.T., H.T., and E.D. critically revised the manuscript; all authors reviewed and approved the manuscript and agreed to be accountable for the work.

## Funding

The study was funded by The Procter & Gamble Company. Medical writing assistance was funded by The Procter & Gamble Company.

## Ethics Statement

Ethical approval for this research was obtained from Hadassah Medical Center Institutional Review Board (IRB) committee (Jerusalem, 91 120, Israel; Ref: HMO 0401–24).

## Consent

Each child and a parent or legal guardian provided written informed consent before enrolment in the study.

## Conflicts of Interest

Dr. Ccahuana‐Vasquez, Dr. Timm, and Dr. Grender are employees of Procter & Gamble, the study sponsor. Dr. Zini, Dr. Teitelbaum, Ms. Zecharyahu, and Dr. Davidovich have received grants from Procter & Gamble.

## Data Availability

The datasets generated and analyzed during the current study are not publicly available as they are considered proprietary, but may be available from the corresponding author upon reasonable request.

## References

[1] M. Kazeminia , A. Abdi , S. Shohaimi , et al., “Dental Caries in Primary and Permanent Teeth in Children's Worldwide, 1995 to 2019: A Systematic Review and Meta‐Analysis,” Head & Face Medicine 16, no. 1 (2020): 22, 10.1186/s13005-020-00237-z. 33023617 PMC7541284

[2] World Health Organization , “The Global Health Observatory. Prevalence of untreated caries of deciduous teeth in children 1–9 years 2023,” accessed July 10, 2025, https://www.who.int/data/gho/data/indicators/indicator‐details/GHO/prevalence‐of‐untreated‐caries‐of‐deciduous‐teeth‐in‐children‐1‐9‐years.

[3] American Dental Association Foundation , “Give Kids a Smile Toolkit. Healthy Habits Handout 2025,” accessed December 8, 2025, https://www.adafoundation.org/‐/media/project/ada‐organization/ada/adaf/files/2025_ada_gkas_education_card_proof.pdf?rev=192969c0ae8942aeb4a5d052129ca906&hash=0CA1E7417F32F98CBBB07E4BC3F7B3E8.

[4] S. O. Griffin , L. Wei , S. Naavaal , and E. Fleming , “The Contribution of Different Permanent Tooth Types to Untreated Caries: Implications for Public Health Surveillance and Prevention,” Journal of the American Dental Association (1939) 152, no. 4 (2021): 269–276.e2. 33775286 10.1016/j.adaj.2021.01.003 PMC10026557

[5] J. M. Broadbent , W. M. Thomson , J. V. Boyens , and R. Poulton , “Dental Plaque and Oral Health During the First 32 Years of Life,” Journal of the American Dental Association (1939) 142 (2011): 415–426. 21454848 10.14219/jada.archive.2011.0197

[6] Fact.MR , “Kids Toothbrush Market,” accessed August 14, 2025, https://www.factmr.com/report/kids‐toothbrush‐market#:~:text=Sales%20of%20Which%20Type%20of,of%20electric%20toothbrushes%20going%20forward.

[7] E. Davidovich , R. A. Ccahuana‐Vasquez , H. Timm , J. Grender , P. Cunningham , and A. Zini , “Randomised Clinical Study of Plaque Removal Efficacy of a Power Toothbrush in a Paediatric Population,” International Journal of Paediatric Dentistry 27 (2017): 558–567. 28494116 10.1111/ipd.12298

[8] E. Davidovich , R. A. Ccahuana‐Vasquez , H. Timm , J. Grender , and A. Zini , “Randomised Clinical Study of Plaque Removal Efficacy of an Electric Toothbrush in Primary and Mixed Dentition,” International Journal of Paediatric Dentistry 31 (2021): 657–663. 33225464 10.1111/ipd.12753 PMC10015989

[9] E. Davidovich , R. A. Ccahuana‐Vasquez , J. Grender , H. Timm , H. Gonen , and A. Zini , “A 4‐Week Randomized Controlled Trial Evaluating Plaque and Gingivitis Effects of an Electric Toothbrush in a Paediatric Population,” International Journal of Paediatric Dentistry 34, no. 3 (2024): 246–255. 37864381 10.1111/ipd.13130

[10] E. Davidovich , S. Shafir , B. Shay , and A. Zini , “Plaque Removal by a Powered Toothbrush Versus a Manual Toothbrush in Children: A Systematic Review and Meta‐Analysis,” Pediatric Dentistry 42 (2020): 280–287. 32847667

[11] F. Dağdeviren , G. A. F. Van der Weijden , C. P. L. Zijlstra , and D. E. Slot , “The Effectiveness of Power Versus Manual Toothbrushes on Plaque Removal and Gingival Health in Children‐A Systematic Review and Meta‐Analysis,” International Journal of Dental Hygiene 23 (2025): 682–702, 10.1111/idh.12915. 40739767 PMC12516003

[12] E. Davidovich , J. Grender , and A. Zini , “Factors Associated With Dental Plaque, Gingivitis, and Caries in a Pediatric Population: A Records‐Based Cross‐Sectional Study,” International Journal of Environmental Research and Public Health 17, no. 22 (2020): 8595. 33228082 10.3390/ijerph17228595 PMC7699320

[13] G. A. Quigley and J. W. Hein , “Comparative Cleansing Efficiency of Manual and Power Brushing,” Journal of the American Dental Association (1939) 65 (1962): 26–29. 14489483 10.14219/jada.archive.1962.0184

[14] S. Turesky , N. D. Gilmore , and I. Glickman , “Reduced Plaque Formation by the Chloromethyl Analogue of Victamine C,” Journal of Periodontology 41 (1970): 41–43. 5264376 10.1902/jop.1970.41.41.41

[15] M. Yaacob , H. Worthington , S. Deacon , et al., “Powered Versus Manual Toothbrushing for Oral Health,” Cochrane Database of Systematic Reviews 2014 (2014): CD002281. 24934383 10.1002/14651858.CD002281.pub3 PMC7133541

[16] Y. Zou , J. Grender , R. Adam , and L. Levin , “A Meta‐Analysis Comparing Toothbrush Technologies on Gingivitis and Plaque,” International Dental Journal 74 (2024): 146–156. 37481415 10.1016/j.identj.2023.06.009 PMC10829363

[17] T. A. Elkerbout , D. E. Slot , N. A. M. Rosema , and G. A. Van der Weijden , “How Effective Is a Powered Toothbrush as Compared to a Manual Toothbrush? A Systematic Review and Meta‐Analysis of Single Brushing Exercises,” International Journal of Dental Hygiene 18, no. 1 (2020): 17–26. 31050195 10.1111/idh.12401 PMC7004084

[18] T. M. J. A. Thomassen , F. G. A. Van der Weijden , and D. E. Slot , “The Efficacy of Powered Toothbrushes: A Systematic Review and Network Meta‐Analysis,” International Journal of Dental Hygiene 20, no. 1 (2022): 3–17. 34877772 10.1111/idh.12563 PMC9303421

[19] R. Adam , J. Grender , H. Timm , J. Qaqish , and C. R. Goyal , “A Randomized Controlled Trial Evaluating a Novel Oscillating‐Rotating Electric Toothbrush Versus a Sonic Toothbrush for Plaque and Gingivitis,” American Journal of Dentistry 38 (2025): 3–8. 40000000

[20] R. Adam , J. Grender , H. Timm , C. R. Goyal , and J. Qaqish , “A 4‐Week Randomized Clinical Trial Evaluating Plaque and Gingivitis Effects of a New Oscillating‐Rotating Electric Toothbrush,” Journal of the American Dental Association (1939) 156, no. 8 (2025): 611–619.e2. 40531078 10.1016/j.adaj.2025.04.015

[21] R. E. Adam , J. M. Grender , H. Timm , J. Qaqish , and C. R. Goyal , “A Randomized Clinical Trial Evaluating Plaque and Gingivitis Effects of an Entry‐Tier Oscillating‐Rotating Electric Toothbrush,” Journal of Dental Hygiene 99 (2025): 6–17. 41022557

[22] A. Bhatti , K. A. Gray‐Burrows , E. Giles , et al., ““Strong Teeth”: The Acceptability of an Early‐Phase Feasibility Trial of an Oral Health Intervention Delivered by Dental Teams to Parents of Young Children,” BMC Oral Health 21, no. 1 (2021): 138. 33743641 10.1186/s12903-021-01444-z PMC7980542

[23] A. Sujlanna and P. K. Pannu , “Family Related Factors Associated With Caries Prevalence in the Primary Dentition of Five‐Year‐Old Children,” Journal of the Indian Society of Pedodontics and Preventive Dentistry 33, no. 2 (2015): 83–88. 25872623 10.4103/0970-4388.155108

